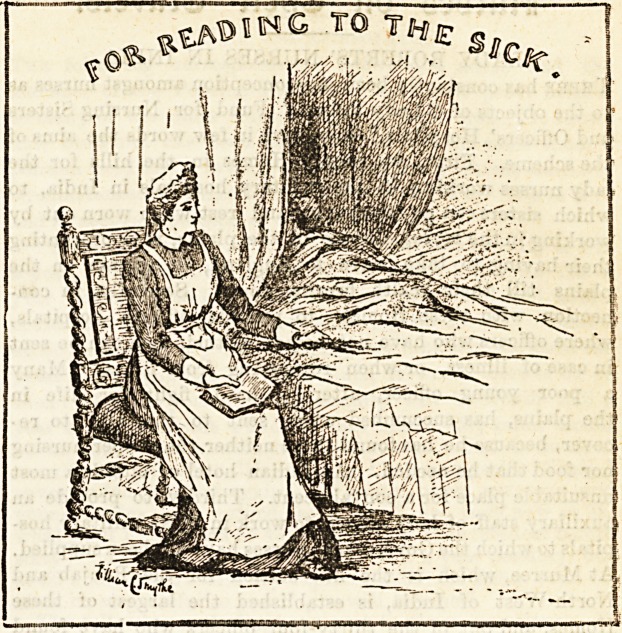# Extra Supplement—The Nursing Mirror

**Published:** 1891-02-07

**Authors:** 


Being the Extra Nursing Supplement of "The Hospital" Newspaper.

Contributions for this Supplement should be addressed to the Editor, The Hospital, 140, Strand, London, W.C., and should have the word "Nursing" plainly written in left-hand top corner of the envelope.

### En Passant.

FROME NURSES' HOME.—The annual meeting of this Home was held last month, when Mrs. Duckworth read the report. The private nurses have attended 58 cases, and the district nurses 172 cases during the past year. The accounts are satisfactory. The Home has lately removed into more commodious premises on the South Parade.

PENSION FUND PRESENTATION.—Nurses who have been in communication with Miss Hale, of Rotherham, and with Miss Belcher, of London, on this subject, are informed that it has been decided that the presentation shall take place about the middle of March. Full particulars will be published so soon as the actual date is fixed.

HELPING ONE ANOTHER.—Our many readers who have visited the Brassey Holiday Home at St. Leonards will not be surprised to hear that the heads of that Home have in a sisterly manner received, free of charge, a nurse sadly in need of a change and utterly without means. We sometimes wish we could take some of the First Thousand to see the cases that the Junius S. Morgan Benevolent Fund is helping; then they would know at last what nurses can do for one another. Take one case only. A nurse with cancer is receiving 7s. a-week from the Junius S. Morgan Fund. She has been a nurse for ten years, but never been able to save. She is now living with her sister, who earns 16s. a-week as a machinist. Without the extra 7s. these two would be obliged to part. It is not possible to go into details of these cases, but the First Thousand ought to know that the Fund is already of the greatest use.

SHORT ITEMS.—Miss Florence Nightingale has returned to town after spending six months in Buckinghamshire.—*Work and Leisure* for February contains a review of last year's progress in nursing matters.—Nurse Petit has resigned her post as midwife to the Western General Dispensary, and is returning to New York.—The post of matron to the Friendly Societies' Home, Grange-over-Sands, has been filled; testimonials will be returned.—The Epsom District Nursing Society has completed three years' good work; it is losing its indefatigable Hon. Secretary, Mrs. Robertson Rodger.—Miss Amy Petier, of New York, has been appointed nurse at the Larnica Hospital, Cyprus.—Lady Alexandra Leveson Gower is very ill; she has never recovered the strain of her nursing experiences of last year.—Lady Macpherson Grant is lecturing on "Home Nursing" at Bridge of Aven.—The Red Cross Sisters of Dublin have received £10 towards the formation of a library for nurses.

THE BEATING OF PATIENTS.—This is not a question of massage and "tapotement," it is a remnant of the old Sairey Gamp days lingering in our midst, and deserving to be shown up for the sake of true nurses, though such a subject can only be approached with shame. Nurse Selina Taylor, of the Keighley Workhouse Infirmary, acknowledged to the Committee who called her conduct in question that she struck a patient with her open hand. The question of a trained nurse for Cockermouth Infirmary having been brought forward, Mr. Herbert said that a nurse at Whitehaven had "taken a stick" to the patients; the Chairman said that one of the untrained nurses of the Cockermouth Infirmary had assaulted a lunatic with a poker. It is noteworthy that all these three stories refer to Workhouse Infirmaries, and we think they reflect chiefly on the Guardians who chose such bad nurses. Would that the Workhouse Infirmary Nursing Association had more money and more power!

COVENTRY NURSING ASSOCIATION.—The annual meeting of this Association was held on January 15th, Mr. E. Petre presiding. A third nurse has been at work during the past year, and the Society proposes to affiliate with the Queen's Jubilee Institute. The accounts show a small deficit. Dr. Milner Moore spoke very heartily in favour of the Association, and in truth it largely owes its success to his hearty support.

ANOTHER FEVER HOSPITAL SCANDAL.—The Rev. S. Bond has brought certain charges against the Bolton Fever Hospital, notably that his child while there was not kept clean, and was permitted to see rude actions. At first a sub-committee considered the charges, and did not hold them to be proved; but last week the General Committee carried the following resolution: "That information having come to the knowledge of this Committee since the presentation of the Hospital Sub-Committee's report in relation to the child of the Rev. S. Bond, which tend to show material defects in the administrative work of the Borough Fever Hospital, this Committee resolves that means be at once taken to place such administration on a more satisfactory footing, and furthermore regrets that Mr. Bond should have had occasion to make any complaint in respect to the treatment of his child." We expect that the Committee have been influenced by the fact that this is not the first scandal at Bolton Fever Hospital, and also by the ill-advised letters two nurses sent to the local papers. We congratulate the Committee on having at last screwed up their courage to have a clean sweep.

HOSPITAL SCANDALS.—Nothing is more disgraceful to modern journalism than the attempt of one or two papers to live by advertising themselves as the exposers of hospital scandals. A correspondent has called our attention to the fact that one such paper has been advertising in our pages, and we have promptly suppressed the advertisement. The present so-called scandal is the illness of nineteen of the St. Bartholomew nurses. Once more, it is not the nurses who complain; it is outsiders who love to rake up charges against philanthropic institutions. No rôle in life is easier than to look on and to find fault; no rôle is more contemptible. The amount of illness amongst the St. Bartholomew nurses is greatly to be regretted. We referred to it during December when we noted the illness of Miss Stewart, and we spoke about it last week. We have made enquiries, and find that the nurses on duty in the diphtheria ward receive soup and wine extra; the only possible addition to the arrangements for their health which we could suggest would be extra time off duty. All the nurses at St. Bartholomew's would be the better for more time off duty, but especially those in charge of contagious cases. Unless we are mistaken, it would be wiser to invest more power in the Matron. Where reforms—even petty reforms—have to pass through endless hands, and are hampered by red-tape, it takes longer than is right to secure redress. It is almost like a retribution that the hospital which was held up at the Lords' Committee as a glorious contrast to the London Hospital should itself now be suffering from an unjust and bitter attack; only, as is common in this world, retribution has fallen on the wrong persons. Our sympathy is very strongly with the authorities of St. Bartholomew's in their present trying position; and once more we glory in the fact that the nurses themselves are loyal to their *alma mater*, and that the charges receive no confirmation from those within the walls.

### Lectures on Surgical Ward Work and Nursing.

By Alexander Miles, M.B. (Edin.), C.M., F.R.C.S.E.

### LECTURE XII.—MANAGEMENT OF SURGICAL OPERATIONS.

After what I have already said of the importance of antiseptic details in connection with surgical dressings, it would be needless and time-wasting repetition to say more in speaking here of operations. Your duties in relation to operations are by no means the least important you have to perform, and I need scarcely impress upon you the necessity of making yourselves thoroughly conversant with them.

Operating Theatre.—This room should be large, well ventilated, and, if possible, lighted from the roof as well as by windows. It is a great advantage to have a theatre which can be used for operations and for nothing else. Your first duty is to see to the sanitary condition of the room in which the operation is to be performed. Early in the morning the floor, walls, and, if necessary, the roof, should be rubbed over with a damp cloth, the windows widely opened, and the theatre thoroughly ventilated. This having been done the fire is kindled, and proper ventilation secured and maintained. If it be particularly important that the air be thoroughly aseptic, for example, in cases in which the peritoneal cavity is opened, one or two carbolic spray engines may be kept working for an hour or two before beginning the operation. The temperature of the room should be about 65 deg. F., and there must be no draughts. The further arrangement of the theatre will depend greatly on circumstances, such as the size and shape of the room, the arrangement of the light, the number of assistants available, and the presence or not of spectators, and must consequently be left to the good sense and management of the nurse. For purposes of description, however, I shall try to indicate what I have seen to be a satisfactory arrangement, in a large teaching hospital, where there was no lack of assistance, and where there were usually a considerable number of spectators at operations. In the centre of the semi-circular theatre stands the operating table. This may be either a fixture, or, better still, arranged on wheels so that the patient may be comfortably placed on the table in the ward, wheeled to the theatre, and the administration of chloroform commenced at once. This means of transferring the patient from the ward to the theatre is a great improvement on the stretchers or basket. A most convenient table is in use in several of the wards of the Royal Infirmary, Edinburgh. It consists of a light but strong table, mounted on light carriage wheels with moderately strong springs. The wheels are furnished with indiarubber tyres, which enables them to run smoothly and quietly. The legs are similarly finished. The table is so balanced that the weight of the patient is never sufficient to cause it to tilt up, and his head being towards the wheel end it can very readily be depressed should any accident occur during the administration of the anæsthetic. The handles by which the table is wheeled are supplied with hinges, so that during the operation they can be folded out of the way, or they may be made to telescope along the sides. Running along the sides are two brass bars, on which broad leather belts for securing the patient slide. The table is 6 ft. long, about 3 ft. high, and 1¾ ft. broad. It may either be bare, or have a thin, firm, hair cushion.

To "Set the Operating Table."—Spread on it, or on the cushion, as the case may be, a thick sheet of indiarubber, and over this a double fold of blanket. On this the patient lies, and he is covered with one or two blankets as may be necessary. Under his head is put a firm pillow filled with hair, feathers being too soft. The pillow should have a macintosh pillow-slip under the linen one to prevent blood or lotion spoiling it. It will be found convenient to have a broad belt passing over the patient's chest and another just above his knees, not only to prevent him slipping off the table on the way to the theatre, but also to restrain his struggles while the anæsthetic is being administered. *Caution.*—Let me warn you, however, not to pull the strap round the chest tight lest you should interfere with respiration. It should never be so tight that you cannot pass your arm between it and the chest wall. You will find that a strap when fixed just above a patient's knees prevents struggling very much better than when placed below the joints.

Clove-Hitch Garter.—You will often find it necessary to fix a patient's hands and feet during an operation, and this is very conveniently done by means of knitted "clove-hitch garters" passed round the wrists and ankles, and then tied to the legs of the table. These must always be tied in a reef-bow, *never in a knot*, as it may become necessary to release the limb very quickly at any moment for the purpose of carrying on artificial respiration, for example. These garters are closely knitted with ordinary strong wool, are about seven or eight feet long and about two inches broad. They have the advantage over ordinary cotton bandage, which is generally used, of being soft and slightly elastic, thus not hurting the skin, and when put on as a clove-hitch they cannot possibly become tight enough to strangulate the limb. A "clove-hitch" is made by making two successive loops in the same direction and placing one behind the other.

Side Tables.—On these are placed the lotions, sponges, instruments, &c. Sometimes only one table is available, and everything must be kept on it; but, when possible, it will be found an advantage to have one on each side of the operating table, in order that the lotions, instruments, and sponges, which are necessarily moist and likely to cause a mess, may be kept away from the dry dressings and the anæsthetic tray. These side tables should be about the same size as the operating table, and are conveniently placed about four feet from it.

In describing these we may speak of them as the "Lotion Table" and the "Dressings Table."

1. The Lotion Table.—This may be arranged to suit the tastes and convenience of the nurse whose duty it is to attend it, but she will do well to adopt some systematic arrangement, and to adhere to it, as by doing so she will get into the habit of almost automatically finding anything she may be asked for. However arranged, on this table should be found the following: (1) A Winchester jar carbolic lotion, 1 in 20; (2) a Winchester jar carbolic lotion, 1 in 40; (3) a Winchester jar corrosive sublimate, 1 in 500; (4) a Winchester jar corrosive sublimate, 1 in 1,000; (5) a Winchester jar corrosive sublimate, 1 in 2,000; (6) a Winchester jar boracic acid lotion, saturated; (7) six or eight lotion basins; (8) two kidney-shaped basins; (9) two bleeding cups; (10) six or eight macintoshes (pink jaconette); (11) six or eight carbolised towels; (12) six or eight clean dry towels; (13) a dozen sponges; (14) a pail of hot water, (15) a pail of cold water, (16), a slop-pail, (17) a dirty dressing tray, placed under the table; (18) a nailbrush; (19) a bottle of spirit of turpentine; (20) a quantity of powdered carbonate of soda; (21) a jar containing drainage tubes; (22) a small jar containing safety-pins in 1·20 carbolic; (23) jars containing ligatures and sutures—(*a*) cat-gut, (*b*) whale-gut, (*c*) kangaroo-tendon, (*d*) silk, (*e*) horse-hair, (*f*) silver wire; (24) lead buttons for button sutures; (25) tourniquets—(*a*) Petit's screw tourniquet, (*b*) Foulis' tourniquet, (*c*) Esmark's elastic webbing; (26) instrument tray and tray for artery forceps; (27) syringes—(*a*) Higginson's syringe, (*b*) glass syringe, (*c*) vulcanite syringe, (*d*) brass syringe; (28) box of sawdust, or large tray under operating table.

### Nursing Medals and Certificates.

LIVERPOOL.

In the *Liverpool Mercury* for January 18th, 1862, there is an account of a meeting held in the chapel of the Bluecoat Hospital, the Mayor presiding, to found a training school and home for nurses in connection with the Royal Infirmary. How that small seed has grown and borne fruit is attested by the fact that it is impossible for us to give a list of all the holders of the above medal, for they are numbered by hundreds. Every nurse who joins the Liverpool Nurses' Training School is presented with the medal at the close of her month of trial, and when on duty she wears it pinned on the left breast; it is not a reward of merit, but a badge, and it only becomes the permanent property of those nurses who serve their three years of training and take their certificate.

The Liverpool Workhouse Infirmary certificate, given after three years, runs as follows: "Parish of Liverpool Workhouse Infirmary. Training School for Nurses. This is to certify that ................ has satisfactorily completed a course of ......... years' training as a Hospital Nurse, and that she is considered to be entitled to a Certificate of Efficiency, the estimate of her character and qualifications being as follows: Conduct ............. Qualifications as Nurse in Medical Cases ............ Surgical Cases ............ Midwifery Cases ............" This certificate is signed by the chairman, two medical officers, and the lady superintendent.

The Liverpool Royal Southern Hospital grants nurses the following certificate at the end of one year: "This is to certify that ................ has been a ............ of this Hospital for ...... months and discharged the duties of the office to the satisfaction of the Medical Board." This certificate has an engraving of the hospital at the top, and is signed only by the members of the medical board. There is an institution for sending out private nurses in connexion with the hospital.

Midwives and monthly nurses are trained at Brownlow Hill Lying-in Hospital, Liverpool, and are granted certificates; the midwives are also prepared for the London Obstetrical Society's examination.

### Presentations.

On the occasion of Staff-Nurse Love leaving the Edinburgh Royal Infirmary, to take the important post of Night Superintendent in the Glasgow Royal Infirmary, she was made the recipient of many handsome presents. Among them may be mentioned those from the surgeons, the nurses, the patients, and clerks and dressers past and present, the last being a handsome silver afternoon tea service, with tray and inlaid table. Miss Love also received an illuminated address, signed by all those who have come in contact with her during their hospital work, thus showing the high esteem in which she was held.

ROYAL ALBERT EDWARD INFIRMARY, WIGAN.—On Feb. 2nd Miss Marianne Müklenthamp Broadhead, the Assistant Matron, who has been appointed Matron to the Noble's General Hospital, Isle of Man, was presented by the nurses and probationers with a pretty afternoon tea service, as a token of their esteem and regard for her, and as an expression of their kind wishes for her success and happiness in her new appointment. During the time that Miss Broadhead has been Assistant Matron at the Infirmary she has been most unsparing in her efforts to help and teach the assistant nurses and probationers, and much regret is felt among the Infirmary staff generally at the prospect of losing her services.

TO THE YOUNG.

When young men and women leave the shelter of home and the care of their parents to gain a livelihood, it is very important for them to choose good companions, as on them chiefly depends how they spend their lives. If they constantly mix with frivolous people they become worldly, and soon give up all desire to love God. They are carried away by health and high spirits farther than they intend; they listen to those who entice them to folly, and often end in disgrace. It is very much safer and wiser to avoid the idle and vain, for St. Paul tells us "Evil communications corrupt good manners," and David, in the Psalms, says that man is blessed who does not walk in the counsel of the ungodly, but his delight is in the law of the Lord, and in His law will he exercise himself day and night; which means that we should make God's commandments the rule of all our actions, and love to please Him by keeping them. There are so many ways in which we can partially break the whole ten without falling into the lowest depths of sin, that it is very helpful to have good friends who themselves keep in the straight and narrow way.

You are now laid by for a little time and prevented by sickness from following your usual employments. Let me ask you to use this leisure in thinking over your past life, and finding out whether you have chosen your friends wisely and well. It is a matter for great thankfulness if you have, but if not, determine at once to break with them as soon as possible. It may be hard at first, but pray to Christ to help you. You cannot manage it in your own strength, but the Lord will make you brave to resist temptation and the scorn. Jesus is the best friend any one can have, He is the friend that sticketh closer than a brother. He is even now standing knocking at the door of your hearts, wanting to come in. Do not send Him away. Open your heart and ask Him to be your comrade. Where He enters He is a most pleasant guest. Do not be afraid your life will be dull and gloomy, with Jesus for your friend.

Why should we fear youth's draught of joy
 If pure would sparkle less?
Why should the cup the sooner cloy
 Which God hath deigned to bless?

No, the world's pleasure will soon cease to please and worldly companions fail to amuse, but the true Friend will never desert you but will satisfy you with good things making your youth to renew like the eagle's.

### Nurses on Their Travels.

### LADY ROBERTS' NURSES IN INDIA.

There has constantly been misconception amongst nurses as to the objects of "Lady Roberts' Fund for Nursing Sisters and Officers' Hospitals," so we give in few words the aims of the scheme. First, to supply Homes in the hills for the lady nurses working in the military hospitals in India, to which sisters can go for change and rest when worn out by working in the trying climate of the plains, thus preventing their having to take leave to England, or remain on the plains till their health breaks down. Secondly, in connection with these Homes, to provide officers' hospitals, where officers who have not homes of their own can be sent in case of illness, or when recovering from illness. Many a poor young officer, after a hard fight for life in the plains, has succumbed when sent to the hills to recover, because he has found there neither the proper nursing nor food that he needed. An Indian hotel or club is a most unsuitable place for a convalescent. Thirdly, to provide an auxiliary staff of lady nurses to work in those military hospitals to which the Government nurses have not been supplied. At Murree, which is the hill station for the Punjab and North-West of India, is established the largest of these Homes, and out of the thirty-four officers who have found shelter there in times of illness, chiefly when suffering from typhoid fever, only two have died. One was a surgeon moved in much too late (being in the third week of enteric fever), who died shortly after his admittance, and the other a young officer of the Seaforth Highlanders, who was so injured by a fall over a cliff while out shooting that he died within thirty-six hours.

A smaller home has been built at Kasauli, and one at Wellington, in the Madras Presidency; and last year a Home was built at Quetta, a town which has become a very large cantonment for European troops.

That the Government has recognised the necessity for these officers' hospitals is proved by the fact that the patients are allowed to be attended by the depôt surgeons, and that medicines are supplied gratis from the Government dispensaries. The officers are charged, if under the rank of captain, five rupees a day; if over that rank, six rupees a day. This allows the youngest subaltern to have skilled nursing care, and all the expensive requirements of severe illness, and yet live well within his pay. The fees charged cover the actual expenses, but the nurses' salaries, building expenses, &c., all come out of Lady Roberts' Fund.

The auxiliary staff of nurses is for the soldiers; they receive their passage money, outfit, and a good salary. They are sometimes sent out to nurse officers in their own homes.

From the above particulars two things are evident: That Lady Roberts' Fund does a good work, and that the expenses must be very heavy. Up to the present time, after meeting all expenses, Lady Roberts has invested 114,000 rupees, for she is well supported by the army at large while she is amongst them. But Lady Roberts knows that her personal influence, energy, and interest cannot be looked for in others, and her great desire is to leave enough money invested when she leaves India to prevent the work collapsing after the withdrawal of her personal support.

### Appointment.

[It is requested that successful candidates will send a copy of their applications and testimonials, with date of election, to The Editor, The Lodge, Porchester Square, W.]

Isle of Man Hospital.—Miss M. Broadhead, of the Royal Albert Edward Infirmary, Wigan, was duly appointed matron of Noble's Isle of Man General Hospital.

### The Lords' Committee.

Last week the Select Committee on Metropolitan Charities recommenced their sittings, before a diminished audience. On the Monday Guy's Hospital came up for judgment, and Mr. Lushington was examined. He stated that he had been fifteen years treasurer at Guy's, and during that time progress had been notable, especially in the nursing department. With regard to the hospital accommodation they had 600 beds, but, owing to want of funds, about 100 beds were now vacant. They always kept about 50 beds vacant, so as to be ready for any serious outbreak of cholera, dysentery, and so on, or for a serious railway accident. There had been very few complaints as to the food supplied to the nursing staff, and those had been with regard to the cooking rather than with regard to quantity or quality. The nurses were expected to be out of their dormitories at twenty minutes past seven, they breakfasted at half-past seven, and they entered the wards at eight o'clock; they dined in two batches, the first dinner was at 11.30 to 12, and the second was at 12 to 12.30, half an hour being allowed for dinner. They had tea at 4.30 to 5, and 5 to 5.30. They had supper at 8.30 to 9, and 9 to 9.30, and they left their wards at 9.45. The nurses themselves were disposed to curtail their time for meals. The night nurses came on duty at 9.30. They had breakfast at 9.10 p.m., and they left their wards at 10.30 the following morning, so that for the best part of an hour the whole staff was on duty at the beginning of the day, when it was very important to have them. The nurses usually made two light meals at night in the wards, one about midnight, and one at about five o'clock in the morning; they did not have any beer in the wards, and the whole of the milk came from the hospital estates. As to the appointment of the hospital staff, the sisters were appointed by himself on the recommendation of the matron, and chosen from among the lady pupils who had had a year's training, and knew all about the hospital and the hospital duties. The nurses were chosen from amongst the probationers who had eighteen months' training, and during their term of probationership they were subject to dismissal by the matron. For the first month they had the option of terminating their engagement if they thought proper, but after the first month they signed a paper saying that they would serve the hospital for three years.

Dr. Perry, warden, gave particulars of the medical college, and spoke well in favour of the much-abused medical student. The college was a commercial speculation, and no part of the hospital money was used for it.

On the Thursday St. Bartholomew's Hospital was made the subject of discussion, and apparently excited more widespread interest.

Mr. W. H. Cross, clerk to St. Bartholomew's Hospital, was examined by the Chairman, and said he had held his present position 24 years, the salary attached to the office being £1,000 a-year, with a house and gas. He described the management of the hospital by the court of governors, treasurer, and almoners, and the several duties of the staff. The hospital possessed about 11,000 acres of property in various English counties, the greater part being in Essex. The hospital includes the whole of the parish of St. Bartholomew-the-Less, and also jutted into other parishes, in respect of which, for residences, they paid £1,186 in rates. As to the manner in which hospital accounts were prepared, he did not see very much advantage in the assimilation of the accounts of the various London hospitals. Last year they admitted into the hospital 140 patients suffering from diphtheria in all stages of the disease, which was double the number received during the previous year. Twenty-three of the nurses contracted the disease. They had lost one nurse from typhoid fever, but none from diphtheria. There was no reason to suppose that there was anything wrong with the quarters given to the nurses, but the surveyor had been instructed to make a complete examination of the quarters throughout the hospital. They had removed the nurses from some cubicles because they found that one nurse had scarlet fever, and it was thought advisable to disinfect the whole place, and the nurses were moved into another house, which was in course of enlargement for the purpose of putting them into. The treasurer's house, which that officer had not occupied for some years, was just now finished for an additional staff of night nurses.

Dr. Moore spoke up for his medical students and for the English system of medical education. He refused to acknowledge that the out-patients were seen too rapidly or that the out-patient department was abused. He gave his listeners the impression that he was well satisfied with things as they are; but he concluded with a little joke at the expense of special hospitals, saying that in special hospitals for diseases of the chest you always found a few patients whose lungs were not affected, proving that the staff found it dull to be restricted to one disease.

On February 2nd, Mr. Robert Brass and Dr. Ord were examined with regard to St. Thomas's. Save for two or three reporters the room was empty. Mr. Brass spoke of the want of hospital accommodation for South London; they had often to send patients away; they could fill the hospital if it were twice its present size. Dr. Ord gave evidence in favour of the present system of medical education, and against the scheme of a central university. There were 400 students at St. Thomas's; they paid 125 guineas for the whole curriculum. Last year the fees produced £8,000; the expenses were £3,000. Dr. Ord stated that he did not consider special hospitals necessary for cancer or for consumption, but that he thought the ophthalmic hospitals very valuable. Special hospitals had increased very rapidly of late years.

### Everybody's Opinion.

*[Correspondence on all subjects is invited, but we cannot in any way be responsible for the opinions expressed by our correspondents. No communications can be entertained if the name and address of the correspondent is not given, or unless one side of the paper only be written on.]*

### IN DARKEST ENGLAND.

NURSE CLARE writes: "In reading General Booth's book, 'Darkest England,' I think 'Brisbane' ought to be thanked for her testimony respecting the treatment of unfortunate women in our hospitals. I have worked for many years in one of England's largest infirmaries, and know of many cases admitted direct from the street, and can confidently say they were not treated as Mr. Booth would have the public believe. Far from it; they were treated with the greatest delicacy, and exactly as other cases. Never once did I hear of them being taunted with their unfortunate life, or made to feel their position in any way. 'General' Booth must indeed be in darkness as to hospital administration, the nursing discipline, and the infused Christian teaching of the Mother Church, which bids us "Be kind one to another, tender-hearted, forgiving one another, even as God for Christ's sake hath forgiven us."

### CONSUMPTION CURES.

"IGNORAMUS" writes: "Having seen in the daily papers that another cure has been discovered by two French doctors I am anxious to know the results of their experiments. Will the person who has goat's blood injected into his veins retain his own instincts, or will he become like an izard or a chamois, and skip upon the mountains?"

### National Pension Fund for Nurses.

### A HELP AND CONSOLATION.

THE following correspondence between a nurse and the manager of the Pension Fund will no doubt prove helpful to many of the older nurses at the present time. It shows how the Fund has been made, at one and the same time, a pension fund and a savings bank, and many nurses similarly circumstanced may like to follow the example of "Nurse A.," and so secure a pension at 50 years of age.

"Nurse A." writes: "I own a policy for an annuity of £15 a year, to begin at the age of 60, for which I pay £5 17s. a year under Table B. I am 39 next year, and feel as if I could hold out to 50, but as to enduring till 60—oh! Shall I at 50 be able to pay for bringing my annuity forward ten years? Can you tell me how much it would cost to do so then? And do you think the annuity will be worth £26 a year with the Bonus Fund at that time? My mind would be so much easier with hope before me. One is so tired sometimes, and when life is rough I say to myself, 'Shall I never be independent again? If young house surgeons and young nurses just trained, at 21 or 23, are rude now at 39, what shall I find them at 49 or 59? Can I see freedom before sixty?' I cannot pay more than £5 17s. a year possibly, but at 50 I might part with my capital (£200) for £26 a year. I shall be so grateful to you if you will tell me."

The following reply has been sent to these questions: If you are disposed now, or at the age of fifty, to have your policy adjusted, so that you will be able to enter upon a pension at that date instead of paying premiums up to, and receiving a pension at sixty, the Pension Fund would be happy to meet your wishes. A continuance of your present premium would entitle you to a pension of £5 14s. 8d. at age of 50. To give you an additional pension of £10 at that date, would require a payment down then of £165 17s. 8d. This additional pension of £10 could, however, be secured by an immediate payment of £125 16s. 8d., assuming your age to be 39 next birthday. If at any time you required to withdraw this capital sum, it would be returned with 2½ per cent. interest less cost of administration. Another course would be to enter for sick pay of 10s. a week at once, so that you would then be secure if you are permanently invalided, as in that case you would practically get a retiring allowance of 10s. a week for life. The additional cost under Table F would be £1 per annum. There remains the question of the bonus additions. These are derived from two sources: (a) Profits, and (b) the Donation Bonus Fund. It is too early to say what principle will be adopted in making the distribution, because the Council cannot determine this until the quinquennial valuation at any rate, which will not take place until five years from the date when policies were first accepted. Nothing can therefore be done in this direction until the end of 1893 at the earliest.

### Notes and Queries.

### Queries.

(33) *The Pension Fund.*—Am I eligible for the Pension Fund? I am a paying probationer, and have a little money of my own just now, but it won't last long. Would it be taking charity for me to join the Fund?—*Beta.*

### Answers.

*Nora.*—You will find particulars of all the hospitals in Tasmania, and the nursing arrangements, in THE HOSPITAL for April 5th, 1890.

*A Ratepayer.*—We pay no attention to anonymous contributions. You must send name and address if you desire your communication to be inserted.

*Leo T.*—There is no agent in England; you must write straight to the Secretary, the General Hospital, Brisbane, Queensland.

*Nurse J.*—You will have to join one of the large hospitals for three months as a paying probationer; then if you asked the matron she would doubtless give you all the surgical experience she could. Try the Royal Free Hospital or the London Hospital.

*F. E.*—Beef-tea is, firstly, a stimulant, but it also affords nourishment. The method you describe is the right way of making it. We only answer queries by post when there is urgent reason for so doing.

(32) *Board and Lodging.*—There are plenty of places where Nurse F. can have reasonable board and lodging. She should consult the advertisements in THE HOSPITAL.—I can personally recommend the Nurses' Hotel at 18, Royal Avenue, Chelsea.—*Sister.*

(33) *The Pension Fund.*—You are eligible to join the Pension Fund, and should do so if merely as an example in thrift and forethought to other nurses. The Fund is not a charity. You need not participate in the Bonus Fund if you have scruples.

### Nurse Hilary.

A woman's ward—the "Eleanor" ward of the little hospital of St. Elizabeth, closed in by the wings of London. A long room, down the sides of which were opposing rows of neat beds, and upon almost every one of them lay a type of suffering womanhood. The whiteness of the beds was brightened by two lines of glowing scarlet quilts and two of bright blue jackets, which gave the room the appearance of an animated Union Jack.

It was early afternoon; the morning, with its doctors' and students' rounds, its setting in order, and settling for the day, was over. Dinner was over, too, and cleared away, and an orderly restfulness reigned. Quiet, gay with spring flowers, light with fitful spring sunshine—and yet how many pieces of Life's tragedy were played in that room, how many curious actors in Life's history passed in and out of the polished door!

Nurse Hilary stood at the end of the ward—a tall, girlish figure, not without a touch of quiet dignity, dark-haired, with dark grey, earnest eyes, and a mouth whose curves expressed many things. Not at all a pretty face, but a good and fine face, giving one the idea of much deep feeling and strength of character underlying its pleasant outlook. The almost Puritan simplicity of the dark blue uniform, with its snow white cap and apron, and deep collar and cuffs, seemed to harmonize with her simple straightforward manner.

A stout elderly visitor had just come in, and was busily talking to her, unpacking a basket of grapes, and nodding to the patients all at once. This was Lady Beckett, the founder of the Eleanor ward, and evidently a welcome visitor, judging from the many smiles that greeted her. Her copper-coloured attire, glittering with many beads, the little flower-bed of staring dandelions and nodding "clocks" that crowned her grey hair and cheerful red-brown face, her violet parasol and navy blue gloves in no wise jarred upon the patients. They remembered her fruit and flowers given so lavishly. It mattered nothing to them—as it did to Society—that her husband had been a self-made, or rather soap-made man, that she sometimes forgot her knife was not a spoon, and unfolded her clean handkerchief carefully in public. They knew of comforts, dainties, and substantial help her riches supplied, and h's misplaced make no difference in the worth of kindly words. She had founded the ward in memory of her daughter—her only child—lost to her, some said through death, others through some sad runaway match; but she herself kept silence, and covered the grave of her sorrow with the flowers of good deeds.

(*To be continued.*)

### A Social Tea.

Last Friday evening the general meeting of the Nurses' Club was held at 12, Buckingham Street, Strand. A large number of members were present. The report was read, and much gratification was felt at the prosperous state of the Institute. Much good work has been accomplished during the past year, and the ever increasing number of members shows how greatly midwives and nurses appreciate the advantages it offers. After the meeting a social tea was given in the pleasant club rooms. Many nurses came from long distances, and a most enjoyable evening was spent. There was something essentially feminine, and, therefore, charming in this way of rushing rapidly through the formalities of a committee meeting, and then descending to tea and gossip.

### Where to Go.

Mrs. Fawcett will lecture to-night (February 7th) at half-past eight, at the Working Men's College, Great Ormond Street, on "The Use of Economies in Education." Admission free.—To-day, at a quarter-past four p.m., Professor Seeley will commence a course of lectures on "The Gravel Beds of the Thames" at Gresham College, Basinghall Street, E C. Tickets for the course 5s. each.—Miss Cobden lectures to the Bermondsey Gladstone Club, 43, Grange Road, S.E., on the evening of February 11th.—On Ash Wednesday a grand sacred concert will be given at eight p.m. at St. James's Hall; balcony seats 3s., and if you go early and get good ones they are the best seats in the hall. Mr. Santley Mr. Edward Lloyd, Mrs. Mary Davies, Madame Antoinette Sterling, and others will sing.—At Niagara Halls, Westminster, there is now on view a panorama of Jerusalem on the day of crucifixion; admission 1s.—It is worth while for nurses to note that admission to the Zoological Gardens is on 6d. on Mondays. The fourth lecture of the Session will be given at the Midwives' Institute and Trained Nurses Club, 12, Buckingham Street, at 7.45 on Friday evening, February 13th, by Dr. Dakin. *Subject*:—"Abnormal conditions of labour and lying-in which can be treated by midwives without the help of a doctor." A few tickets to non-members, price 6d. each, for which early application is requested.

### A Strange Story.

The following extraordinary paragraph has appeared in a local paper:—"Resection of the Head of a Female.—An interesting case was lately performed at Paris. The head of a female having been exposed by a posterior excision, was excised, and then the neck was laid in this cavity. The patient made a good recovery." Can it be possible that this is an unprofessional reading of a resection of the femur?

### Amusements and Relaxation.

### SPECIAL NOTICE TO CORRESPONDENTS.

First quarterly word competition commenced January 3rd, 1891; ends March 28th, 1891.

Competitors can enter for *all* quarterly competitions, but no competitor can take more than *one first prize* or *two* prizes of any kind during the year.

Three prizes of 15s., 10s., 5s., will be given for the largest number of words derived from the words set for dissection.

Proper names, abbreviations, foreign words, words of less than four letters, and repetitions are barred; plurals, and past and present participles of verbs, are allowed. Nuttall's *Standard* dictionary only to be used.

N.B.—Word dissections must be sent in WEEKLY not later than the first post on Thursday to the *Prize Editor*, 140, Strand, W.C., arranged alphabetically, with correct total affixed.

The word for dissection for this, the SIXTH week of the quarter, being "SNOWDROPS."

| Names. | Jan. 29th. | Totals. | Names. | Jan. 29th. | Totals. |
|---|---|---|---|---|---|
| Reynard | — | 77 | Woodbine | — | 25 |
| Reldas | 47 | 193 | Madame B. | — | 25 |
| Tinie | — | 30 | Shakespeare | — | 59 |
| Patience | — | 76 | Smyrna | 22 | 105 |
| Jenny Wren | 45 | 180 | Southwood | 25 | 102 |
| Agamemnon | 47 | 185 | Gipsy Queen | — | 21 |
| Wyamaris | 47 | 185 | Snowball | — | 19 |
| E. C. | 47 | 189 | Rita | — | — |
| Ecila | 44 | 182 | Mortal | — | 16 |
| Hope | 47 | 189 | Nurse Annie | — | 15 |
| M. W. | 47 | 189 | Carmen | — | 11 |
| Qu'appelle | 45 | 187 | Grannie | — | 45 |
| Nil Desperandum | 46 | 183 | Amie | — | 30 |
| Lady Betty | 44 | 174 | M. R. | — | 25 |
| H. A. S. | 24 | 144 | Primrose | — | 24 |
| Sister Jack | — | 62 | Nurse J. S. | 38 | 38 |
| Crystal | 35 | 156 | | | |

### Notice to Correspondents.

N.B.—Each paper must be signed by the author with his or her real name and address. A *nom de plume* may be added if the writer does not desire to be referred to by us by his real name. In the case of all prize-winners, however, the real name and address will be published.

## Figures and Tables

**Figure f1:**
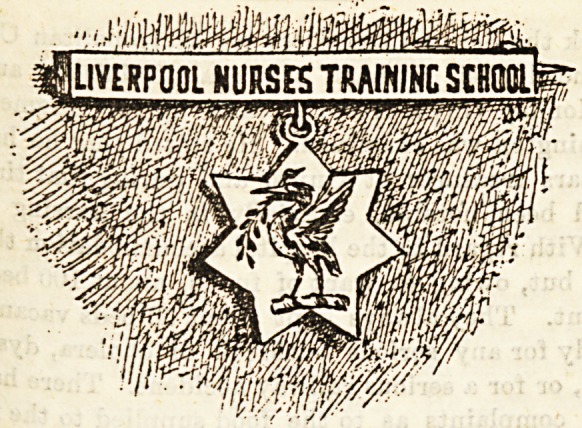


**Figure f2:**